# Divergent clonal evolution of blastic plasmacytoid dendritic cell neoplasm and chronic myelomonocytic leukemia from a shared TET2-mutated origin

**DOI:** 10.1038/s41375-021-01228-y

**Published:** 2021-04-08

**Authors:** Kiran Batta, Hasse M. Bossenbroek, Naveen Pemmaraju, Deepti P. Wilks, Richard Chasty, Mike Dennis, Paul Milne, Matthew Collin, Hannah C. Beird, Justin Taylor, Mrinal M. Patnaik, Catherine A. Cargo, Tim C. P. Somervaille, Daniel H. Wiseman

**Affiliations:** 1 Epigenetics of Haematopoiesis Laboratory, Division of Cancer Sciences, The University of Manchester, Manchester, UK; 2 Department of Leukemia, The University of Texas MD Anderson Cancer Center, Houston, TX USA; 3 Haematological Malignancies Biobank, Manchester Cancer Research Centre, The University of Manchester, Manchester, UK; 4 Department of Haematology, The Christie NHS Foundation Trust, Manchester, UK; 5 Translational and Clinical Research Institute, Newcastle University, Newcastle-upon-Tyne, UK; 6 Northern Centre for Cancer Care, Newcastle-upon-Tyne Hospitals NHS Foundation Trust, Newcastle-upon-Tyne, UK; 7 Department of Genomic Medicine, The University of Texas MD Anderson Cancer Center, Houston, TX USA; 8 Division of Hematology, Sylvester Comprehensive Cancer Center, University of Miami Miller School of Medicine, Miami, FL USA; 9 Division of Hematology, Mayo Clinic, Rochester, MN USA; 10 Haematological Malignancy Diagnostics Service, St James’ University Hospital, Leeds, UK; 11 Leukaemia Biology Laboratory, Cancer Research UK Manchester Institute, The University of Manchester, Manchester, UK

**Keywords:** Cancer genetics, Oncogenesis

## To the Editor:

Blastic plasmacytoid dendritic cell neoplasm (BPDCN) is a rare dermatopathic hematological malignancy derived from plasmacytoid dendritic cell (pDC) precursors. It co-exists with other myeloid malignancies in >20% of cases, and especially frequently with chronic myelomonocytic leukemia (CMML) [1]. Given emerging data for shared clonal origin [2, 3] and supportive clonal pDCs characterizing the CMML microenvironment [4], these diseases might share common biology and therapeutic vulnerabilities. However, their detailed genomic landscape, clonal relationship, and distinctive pathogenesis remain unclear.

A 73-year-old male presented with widespread purpuric lesions over trunk and limbs (Supplementary Fig. 1). Ten years earlier he was diagnosed with low-risk CMML-0, managed throughout by active surveillance. Skin biopsy confirmed BPDCN, with infiltration by moderately proliferative (Ki67 50%) blasts expressing the classic diagnostic triad of CD4, CD56, and CD123 [1], alongside BCL2, CD10, CD33, TdT, and CD99. Blood count revealed a stable monocytosis (1.4 × 10^9^/L) and neutropenia (1.4 × 10^9^/L), but a normal platelet count. Bone marrow (BM) was heavily infiltrated by CMML, with hypercellular, dysplastic myelomonocytic precursors but no blast/promonocyte excess. Flow cytometry of BM revealed 26% CD64+CD14+ monocytes, 1% CD34+ myeloblasts, and 1% neoplastic pDCs (CD4+CD56+CD123+HLA-DR+NG2+), indicating low-level BPDCN involvement. Karyotype was normal. Non-intensive treatment with azacitidine was commenced. After three cycles skin lesions remained unchanged. BM showed regression of CMML but now extensive (>70%) involvement by BPDCN. Azacitidine was discontinued and he was managed with supportive care. Shortly after he developed central visual loss with choroidal infiltrates, indicating central nervous system infiltration. He declined lumbar puncture and died 9 months after BPDCN diagnosis.

**Fig. 1:**
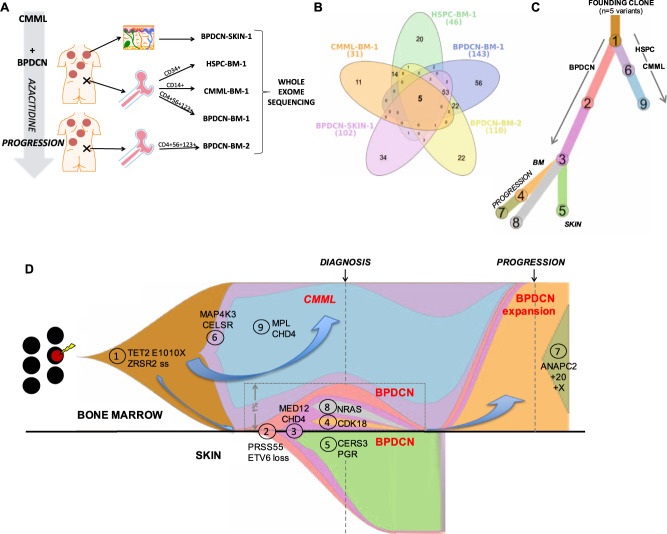
Early divergent clonal evolution of CMML and BPDCN from a shared founding clone. **A** Schematic of experimental setup and samples used for whole exome sequencing from the index patient. **B** Venn diagram showing the distribution of all somatic variants identified across the five sequenced exomes (*n* = 249). **C** Phylogenetic evolution tree for the nine distinct mutation clusters identified by SciClone across the five sequenced exomes; clusters unique to specific compartments are indicated in parentheses. **D** Composite fish plot depicting the clonal architecture and evolution in our index patient. Each color represents the indicated clone and aligns with color scheme in (**C**); cluster numbers were automatically assigned by SciClone and are annotated on the plot. Selected exemplar mutations for each defined cluster are also labeled. Block arrows emphasize the distinct evolution paths toward CMML and BPDCN, and then for the BPDCN marrow expansion at disease progression. For enhanced clarity, the clonal arrangement within the small BPDCN-BM-1 clone (representing 1.08% of total marrow cells by flow cytometry) is expanded within the inset, as indicated. Similarly, the plot for the contemporaneous presentation skin sample (BPDCN-SK-1) is inverted/transposed, to display clonal evolution in this tissue separately from the bone marrow-derived samples (rather than in misleading linear series).

Samples were collected from BM, skin and buccal swab at BPDCN presentation, and from BM following azacitidine. Six samples were prepared for analysis: CD34+ hematopoietic stem/progenitor cells (“HSPC-BM-1”), CD14+ CMML cells (“CMML-BM-1”) and CD4+CD56+CD123+ BPDCN cells (“BPDCN-BM-1”) (from presentation BM by flow sorting); whole skin biopsy from a presenting BPDCN lesion (“BPDCN-SK-1”); sorted BM BPDCN cells post-azacitidine (“BPDCN-BM-2”; CD4+CD56+CD123+); and buccal swab as germline control. All samples underwent whole exome sequencing (WES) on the IonTorrent^TM^ platform (Fig. 1A; Supplementary Methods; Supplementary Table 1; Supplementary Fig. 2).

In total 249 somatic variants (involving 241 genes) were identified across the five non-germline samples, 97 involving coding regions or splice sites with predicted translational consequence (Supplementary Table 2). A *TET2* p.Glu1010Ter nonsense somatic mutation was present in all samples, with mean VAF 50.8% (range 49.8–52.3%). Five somatic mutations were common to all samples, indicating a shared founding clone arising within the HSPC compartment and propagated across both diseases. In addition to the TET2 mutation was a pathogenic splice site mutation in *ZRSR2*, a 5′UTR variant of the E3-ubiquitin ligase *TRIM4*, and passenger (noncoding/synonymous) mutations in *MYO7B* and *THSD7B*.

**Fig. 2:**
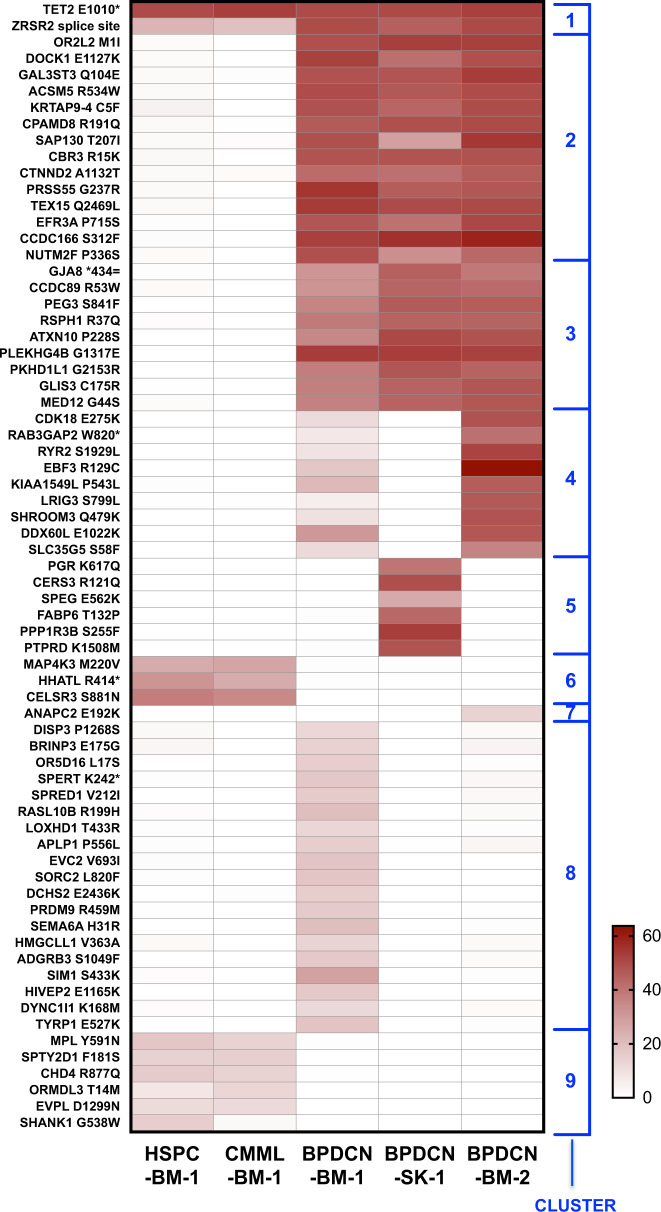
Mutation oncoprint depicting all somatic coding and pathogenic splice site variants (*n* = 98) across each of the five sequenced exomes from the index patient. Variants are arranged according to mutation cluster as determined by ClonEvol/Sciclone (see Fig. 1C, D); color intensity is scaled to represent variant allele frequency for each mutation.

Downstream, the CMML and BPDCN samples displayed markedly different mutation profiles and evolutionary paths, indicating early clonal divergence (Fig. 1B–D; Supplementary Fig. 3) and disease-specific secondary driver lesions (Fig. 2). CMML-BM-1 harbored cancer-associated mutations including *MPL* p.Tyr591Asp, *CHD4* p.Arg877Gln, and *MAP4K3* p.Met220Val; the latter altering the kinase domain of a protein reportedly dysregulated by *TET2* silencing [5]. All CMML-BM-1 mutations were also detectable (at lower VAF) in HSPCs, indicating malignant evolution from the *TET2/ZRSR2*-mutated founding clone within the CD34+ compartment. None were detected in any BPDCN specimen.

By contrast, many more genetic lesions were identified in the BPDCN samples. SciClone resolved nine distinct mutation clusters (Fig. 2). Two large clusters (C2; C3) included mutations displaying high (~40–60%) VAF in all three BPDCN samples, but absent from the HSPC-BM-1 or CMML-BM-1 samples, suggesting a “secondary” BPDCN-initiating event likely outside the CD34 compartment and after pDC precursor egress from BM. These included a hotspot mutation in the putative oncogene *MED12* (p.Gly44Ser), and mutations involving the transcription factor GLIS3 and Rac GTPase regulator DOCK1. SciClone segregated mutation clusters distinct to either BPDCN-SKIN-1 (C5) or BPDCN-BM-1 (C4; C8), indicating subclonal divergence between the cutaneous and BM diseases following recirculation back to BM (Fig. 1C, D). Only C4 was detected in (and dominated) BPDCN-BM-2, highlighting the subclone responsible for BPDCN progression through azacitidine; this included COSMIC-annotated pathogenic mutations involving *CDK18* (p.Glu275Lys) and *EBF3* (p.Arg129Cys). An *NRAS* p.Tyr64Asn mutation was identified in BPDCN-BM-1 only, indicating presence in a clone outcompeted and/or suppressed by azacitidine.

HSPCs contained no high-confidence CNVs. By contrast, all BPDCN samples displayed a significant CNV burden, indicating relative genomic instability. These all harbored a ~5 Mb deletion at chromosome 12p13.31 encompassing 72 genes, including *ETV6* and others with documented roles in DC migration/function (Supplementary Fig. 4). Accordingly, BM cytogenetics post-azacitidine confirmed add(12)(p11.2) and *ETV6* loss by FISH in 56/100 cells (Supplementary Fig. 5). Other CNVs were private to individual samples, indicating later subclonal events (Supplementary Fig. 4). These included several regions of copy gain across chr12 (BPDCN-BM-1); trisomies 2 and 7 (BPDCN-SK-1); chr20 gain and deletions involving the TNF receptor-associated *TRAF7* and MTOR-associated protein *MLST8* (ch16p13.3) (BPDCN-BM-2).

A shared clonal origin of CMML and BPDCN has been proposed previously. Targeted sequencing of a similar patient found *TET2* and *SRSF2* mutations in both BM MNCs and contemporaneous skin biopsy, indicating these as shared early events. *JAK2* was mutated exclusively in BM as a presumed CMML-specific subclonal event [2]. Another case reported WES on unsorted BM MNCs from CMML presentation and later at BPDCN, again demonstrating shared ancestry (including a *TET2* mutation) whilst implicating biallelic *RB1* loss in BPDCN transformation [3]. This implied BPDCN transforming directly from CMML, through acquisition of additional genetic lesions.

Ours is the first study to directly partition CMML and BPDCN from the same compartment, and to sequence (at exome level) sorted subpopulations from different sites and timepoints. We too demonstrate shared initiation, but with remarkably distinct subsequent evolutionary paths in a complex genomic landscape. Again, *TET2* loss-of-function was a shared ancestral event, mirroring its known recurrence in both diseases [6, 7]. For the first time we collated 20 cases of BPDCN co-existing with CMML (or its precursor clonal hematopoietic states), including several previously unpublished (Supplementary Table 3). Remarkably, *TET2* was mutated in 13/13 (100%) of sequenced cases, implying ubiquity in this context and significantly higher frequency versus reported series of unselected BPDCN (*p* = 0.0002; Fisher’s exact test; Supplementary Fig. 6). The divergent phylogenetic paths in our patient epitomize the promiscuous malignant potential of TET2 dysfunction, and the influence of cellular context and secondary lesions in modulating resultant malignant phenotype. This reconciles with observations in murine models, in which *Tet2* loss biased HPSCs toward production of both macrophages *and* DCs, from distinct multipotent progenitor populations, establishing discrete foundations for both diseases [8].

No BPDCN-exclusive mutations were detected in the CD34+ compartment. Thus, although rooted in a preleukemic HSPC, BPDCN transformation occurred via a “secondary” initiation event in a more committed cell, in keeping with its typically CD34-negative immunophenotype [1]. A core set of shared mutations across all BPDCN samples implicates the minor BPDCN-BM-1 population as part of the malignant clone, rather than a benign clonal pDC expansion (a documented feature in CMML-BM [4]). Relative disease proportions in BM and skin suggest extramedullary initiation with subclonal recirculation back to BM. Subsequently one BPDCN subclone outcompeted the other, synchronous with regression of the CMML (also outcompeted, or eradicated by azacitidine).

The most prominent BPDCN-specific lesion was *ETV6* loss, conclusively lacking from CMML cells so precluding detection on initial BM cytogenetics. *ETV6* encodes an ETS family transcription factor that is a key regulator of HSPC function and tumor suppressor. *ETV6* disruption was recently linked to BPDCN [9, 10], with 12p karyotypic abnormalities commonly seen [1]. We also identified *MED12* p.Gly44Ser in BPDCN for the first time, as a novel candidate “secondary” driver mutation. This hotspot mutation occurs frequently (~70–80%) in uterine leiomyoma and breast fibroepithelial tumors (plus their malignant equivalents), 5–10% of chronic lymphocytic leukemias, and sporadically in other cancers [11]. Intriguingly, the highest *MED12* expression in hematopoiesis is reportedly in pDCs (Supplementary Fig. 7), and it is prominently induced upon ex vivo DC activation [12], indicating functional importance in DCs.

*MED12* encodes a kinase module of the transcriptional coactivator Mediator complex, conveying signals from chromatin-bound transcription factors at enhancers through recruitment of RNAPII to promoters. Oncogenic Gly44 mutations impair MED12’s ability to activate CDK8 and CDK19, dysregulating key transcriptional networks [13]. They are tumorigenic in murine models, mediating widespread genomic instability [14]. Notably, *TP53*, *CDKN2A* and *RB1* inactivation, prominent inducers of genomic instability and common in BPDCN, were absent in our patient. MED12 mutations might phenocopy these and represent an alternative route to genomic instability: a fundamental intermediate event in BPDCN pathogenesis. Interestingly *MED12* is X-linked, and not confirmed to escape X-inactivation; if Gly44 substitutions confer tumorigenic gain-of-function as has been proposed [14] this could further contribute to BPDCN’s striking (~3:1) male preponderance.

We investigated whether *MED12* mutations are recurrent in BPDCN. Few suitable existing BPDCN sequencing datasets were identified. We reviewed four published [3, 15] and two unpublished BPDCN-BM exomes, plus eight BM samples sequenced by a 300-gene panel [7]. We also sequenced *MED12* exons 1–2 in 15 archived BM and 9 skin biopsies, validating the mutation in our index patient but finding no others in this limited cohort (Supplementary Fig. 8). Nonetheless, our study highlights this well-described oncogenic mutation as a novel potential BPDCN driver downstream of the apparently ubiquitous *TET2*-mutant foundation for this important clinical association.

## Supplementary information

Supplemental Information

Supplemental Table 2

## References

[1] Khoury JD (2018). Blastic plasmacytoid dendritic cell neoplasm. Curr Hematol Malig Rep.

[2] Brunetti L, Battista VD, Venanzi A, Schiavoni G, Martelli MP, Ascani S (2017). Blastic plasmacytoid dendritic cell neoplasm and chronic myelomonocytic leukemia: a shared clonal origin. Leukemia.

[3] Patnaik MM, Lasho T, Howard M, Finke C, Ketterling RL, Al-Kali A (2018). Biallelic inactivation of the retinoblastoma gene results in transformation of chronic myelomonocytic leukemia to a blastic plasmacytoid dendritic cell neoplasm: shared clonal origins of two aggressive neoplasms. Blood Cancer J.

[4] Lucas N, Duchmann M, Rameau P, Noël F, Michea P, Saada V (2019). Biology and prognostic impact of clonal plasmacytoid dendritic cells in chronic myelomonocytic leukemia. Leukemia.

[5] Berggren DM, Folkvaljon Y, Engvall M, Sundberg J, Lambe M, Antunovic P (2018). Prognostic scoring systems for myelodysplastic syndromes (MDS) in a population‐based setting: a report from the Swedish MDS register. Br J Haematol.

[6] Coltro G, Mangaonkar AA, Lasho TL, Finke CM, Pophali P, Carr R (2020). Clinical, molecular, and prognostic correlates of number, type, and functional localization of TET2 mutations in chronic myelomonocytic leukemia (CMML)—a study of 1084 patients. Leukemia.

[7] Beird HC, Khan M, Wang F, Alfayez M, Cai T, Zhao L (2019). Features of non-activation dendritic state and immune deficiency in blastic plasmacytoid dendritic cell neoplasm (BPDCN). Blood Cancer J.

[8] Ostrander EL, Kramer AC, Mallaney C, Celik H, Koh WK, Fairchild J (2020). Divergent effects of Dnmt3a and Tet2 mutations on hematopoietic progenitor cell fitness. Stem Cell Rep.

[9] Gao NA, Wang X-X, Sun J-R, Yu W-Z, Guo N-J (2015). Blastic plasmacytoid dendritic cell neoplasm with leukemic manifestation and ETV6 gene rearrangement: a case report. Exp Ther Med.

[10] Tang Z, Li Y, Wang W, Yin CC, Tang G, Aung PP (2018). Genomic aberrations involving 12p/ETV6 are highly prevalent in blastic plasmacytoid dendritic cell neoplasms and might represent early clonal events. Leuk Res.

[11] Zhang S, O’Regan R, Xu W (2020). The emerging role of mediator complex subunit 12 in tumorigenesis and response to chemotherapeutics. Cancer.

[12] Costa V, Righelli D, Russo F, Berardinis PD, Angelini C, D’Apice L (2017). Distinct antigen delivery systems induce dendritic cells’ divergent transcriptional response: new insights from a comparative and reproducible computational analysis. Int J Mol Sci.

[13] Park MJ, Shen H, Spaeth JM, Tolvanen JH, Failor C, Knudtson JF (2018). Oncogenic exon 2 mutations in Mediator subunit MED12 disrupt allosteric activation of cyclin C-CDK8/19. J Biol Chem.

[14] Mittal P, Shin Y-h, Yatsenko SA, Castro CA, Surti U, Rajkovic A (2015). Med12 gain-of-function mutation causes leiomyomas and genomic instability. J Clin Investig.

[15] Menezes J, Acquadro F, Wiseman M, Gómez-López G, Salgado RN, Talavera-Casañas JG (2014). Exome sequencing reveals novel and recurrent mutations with clinical impact in blastic plasmacytoid dendritic cell neoplasm. Leukemia.

