# Supplementary material for: Divergent clonal evolution of blastic plasmacytoid dendritic cell neoplasm and chronic myelomonocytic leukemia from a shared TET2-mutated origin
Source: Leukemia. 2021 Apr 8;35(11):3299–303. doi: 10.1038/s41375-021-01228-y (PMC8550946; doi:10.1038/s41375-021-01228-y)
Supplement: Supplementary file 1 — Supplemental Information [file 41375_2021_1228_MOESM1_ESM.pdf]

Supplemental Methods

Patient sampling

The index patient was referred to The Christie NHS Foundation Trust (Manchester, UK) upon diagnosis of BPDCN. Full informed consent was provided to donate blood, bone marrow, germline and skin biopsy samples to the Manchester Cancer Research Centre Haematological Malignancies Biobank, instituted with the approval of the South Manchester Research Ethics committee and the Biobank's scientific sub-committee. Consent was provided for publication of sequencing data, clinical information and images.

Sample preparation

Bone marrow aspirate samples underwent immediate density gradient centrifugation on Lymphoprep to separate the mononuclear cell fraction. This was divided and half processed fresh (same day) for FACS sorting; the remainder was cryopreserved in liquid nitrogen for long-term storage as previously described[1]. Skin biopsy was deposited immediately into saline, kept at 4°C overnight, and genomic DNA extracted the following day.

FACS sorting

BM samples from diagnosis and post azacitidine underwent FACS sorting to enrich the desired subpopulations. Flow cytometry antibodies used were (all mouse anti-human): CD14-Pacific Blue (cat #558121; clone M5E2; BD Pharmingen); CD56-FITC (#555518; clone B159; BD Pharmingen); CD34-PerCP (#345803; clone 8G12; BD Biosciences); CD4-PE (#12-0043; clone RM4-4; eBioscience); CD123-APC (#306012; clone 6H6; BioLegend). Sorts were performed on a BD FACSAria™ II (BD Biosciences, San Jose, CA, USA) instrument. Purity checks were performed where cell numbers permitted. Representative FACS plots depicting the sorting and gating strategy are provided in Supplemental Fig. 2.

Whole exome sequencing

Genomic DNA was extracted using a QIAamp DNA Mini kit (Qiagen, Manchester UK) according to manufacturer instructions. In total six samples were prepared for sequencing: (1) Germline (buccal swab); (2) HSPC-BM-1; (3) CMML-BM-1; (4) BPDCN-BM-1; (5) BPDCN-SK-1; (6) BPDCN-BM-2. Sequencing libraries were manually prepared using the Ion

AmpliSeq Exome RDY kit, according to manufacturer's protocol (ThermoFisher, Paisley, UK). Libraries quantitated by qPCR using the Ion Library TaqMan® Quantitation Kit (#4468802; ThermoFisher) according to manufacturer's protocol. Libraries were diluted to 100 pM and loaded onto Ion 540 chips (one per sample) before sequencing on an Ion GeneStudio S5 instrument (ThermoFisher).

Each somatic exome was sequenced to minimum 200x read depth (Supplemental Table 1). Germline and BPDCN-SK-1 samples were sequenced twice (separate chips; separate runs) and the subsequent BAM files merged into a single sample for each, to ensure sufficient coverage depth, for downstream analyses. Initial data processing was automated using the on board Torrent Suite software. Generated BAM files were uploaded to the Ion Reporter package for further analysis. Somatic mutations and copy number variants (CNVs) were identified and annotated using the Ion Reporter Tumor-Normal pipeline (v5.12) and AmpliSeq Exome Tumor Normal v1 Filter Chain, customised to exclude variants with germline coverage <10X or ≥3% variant reads in germline (allowing for low-level contamination by leukocyte DNA in buccal swab). Variants were manually inspected and excluded if located at amplicon margins, within homopolymer repeats, or otherwise considered artefactual. To robustly determine the distribution and relative VAFs across samples, pairwise comparison of each somatic exome, in all combinations, was performed using the same analytical pipeline. All high confidence somatic variants identified in ≥1 sample were manually inspected across other samples to exclude low-level presence below filtering thresholds. Pathogenicity of confirmed variants was evaluated using Varsome[2], which aggregates computational predictions from 11 different tools. SciClone[3] and ClonEvol[4] were used to cluster all copy neutral somatic SNVs with depth >50x and thereby elaborate clonal architecture.

Screening for other BPDCN/CMML cases

A literature search was performed to collate and review all published instances of BPDCN and CMML co-occurrence. To supplement this, databases were searched at participating institutions for all documented BPDCN cases: The Christie NHS Foundation Trust (Manchester, UK); MD Anderson Cancer Center (Houston, TX, USA); Mayo Clinic (Rochester, MN, USA); Memorial-Sloan Kettering Cancer Center (New York City, NY, USA); Sylvester Comprehensive Cancer Center (Miami, FL, USA); Haematological Malignancy Diagnostic Service (Leeds, UK); and the Human Dendritic Cell Laboratory (Newcastle, UK). Clinical case records were reviewed to identify BPDCN cases with other documented

haematological malignancy diagnoses, and associated demographic, clinical and molecular information.

Supplemental Figures

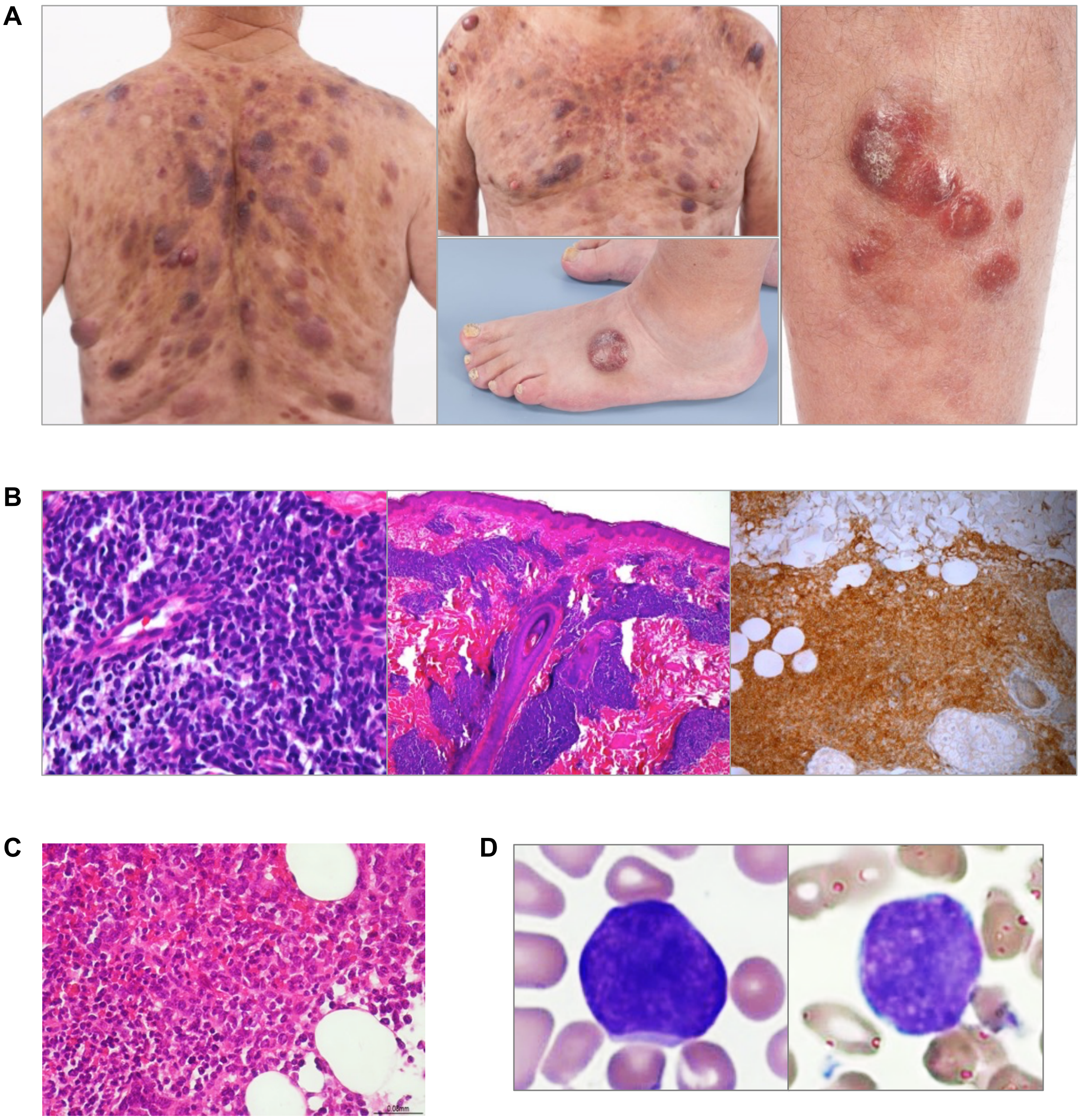

Supplemental Figure 1. A. BPDCN skin lesions in our patient, with violaceous lesions spread extensively across the trunk and lower limbs. **B.** Dermal infiltration by BPDCN in our patient's diagnostic skin biopsy, on H+E (left/middle) and CD123 (right) staining. **C.** Bone marrow trephine histopathology from disease progression after three cycles of azacitidine, showing heavy infiltration by BPDCN. **D.** BPDCN blast morphology from bone marrow aspirate at disease progression after three cycles of azacitidine (same timepoint as C).

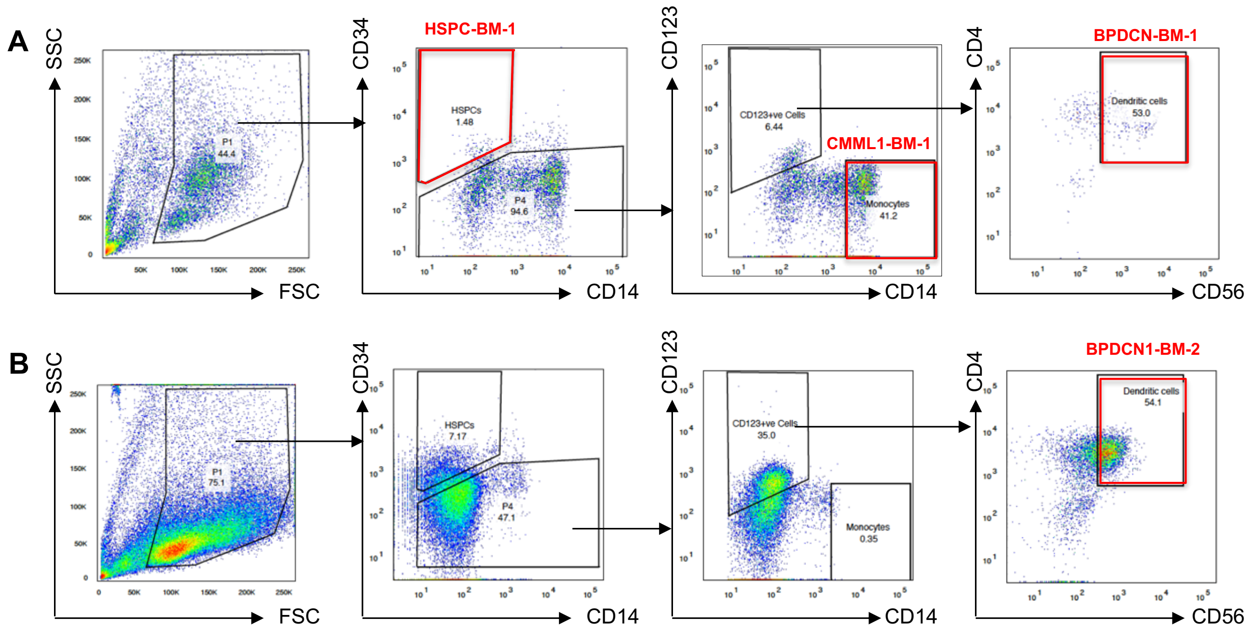

Supplemental Figure 2. Indicative flow sorting strategy employed to separate bone marrow subpopulations for whole exome sequencing from the initial presentation timepoint (**A**) and after three cycles of azacitidine (**B**). The cell populations sorted for genomic DNA extraction and whole exome sequencing are indicated in red.

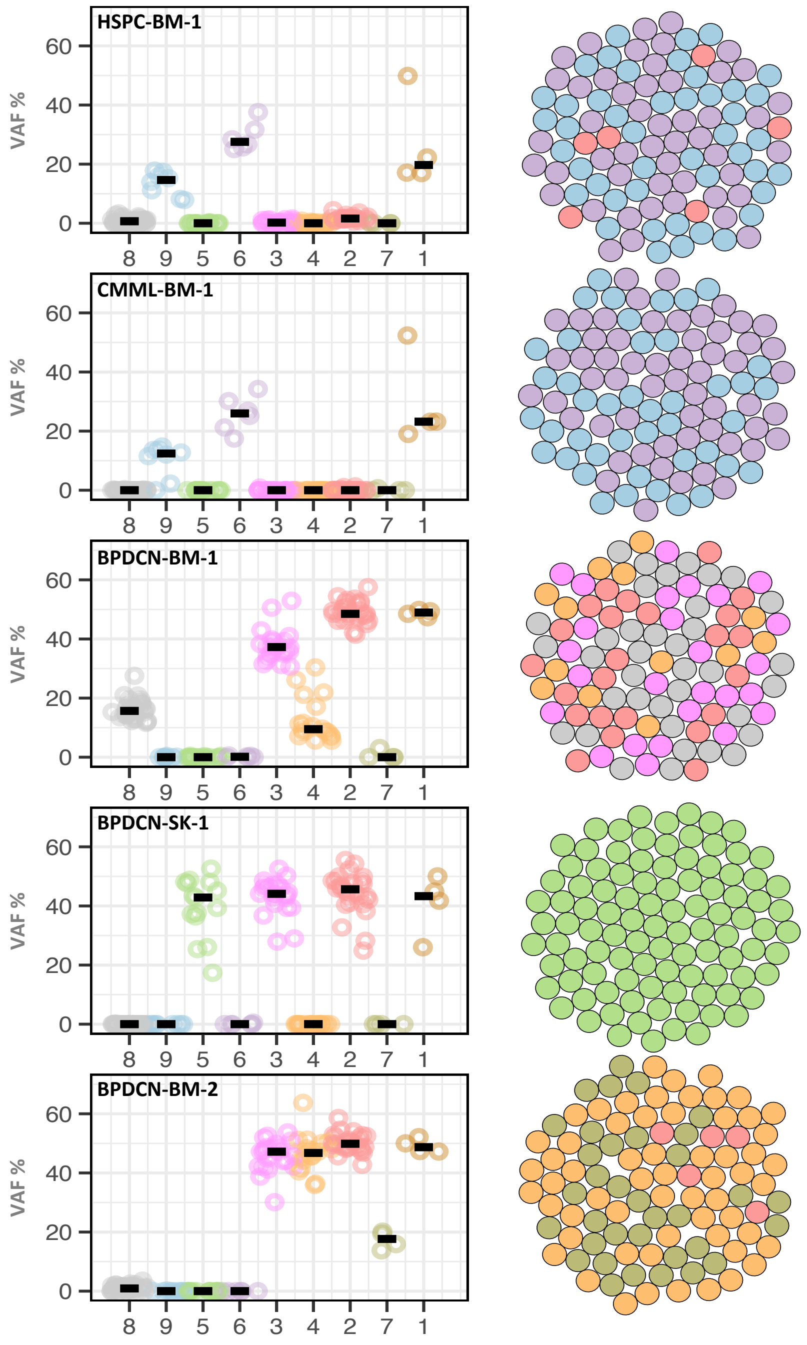

Supplemental Figure 3. Visualization of the composition, sample distribution and variant allele frequencies of constituent mutations for the nine discrete mutation clusters identified by SciClone and modeled by ClonEvol. Shown for each indicated sample are: (left) "Cluster" plots illustrating all identified mutations across the study, arranged by defined cluster, with their respective VAFs within that sample; and (right) "Sphere" plots, as a means to visualize the dominant clonal subpopulations per 100 representative cells for each sample (i.e. illustrating the admixture and approximate percentage of cells belonging to each clone in that sample).

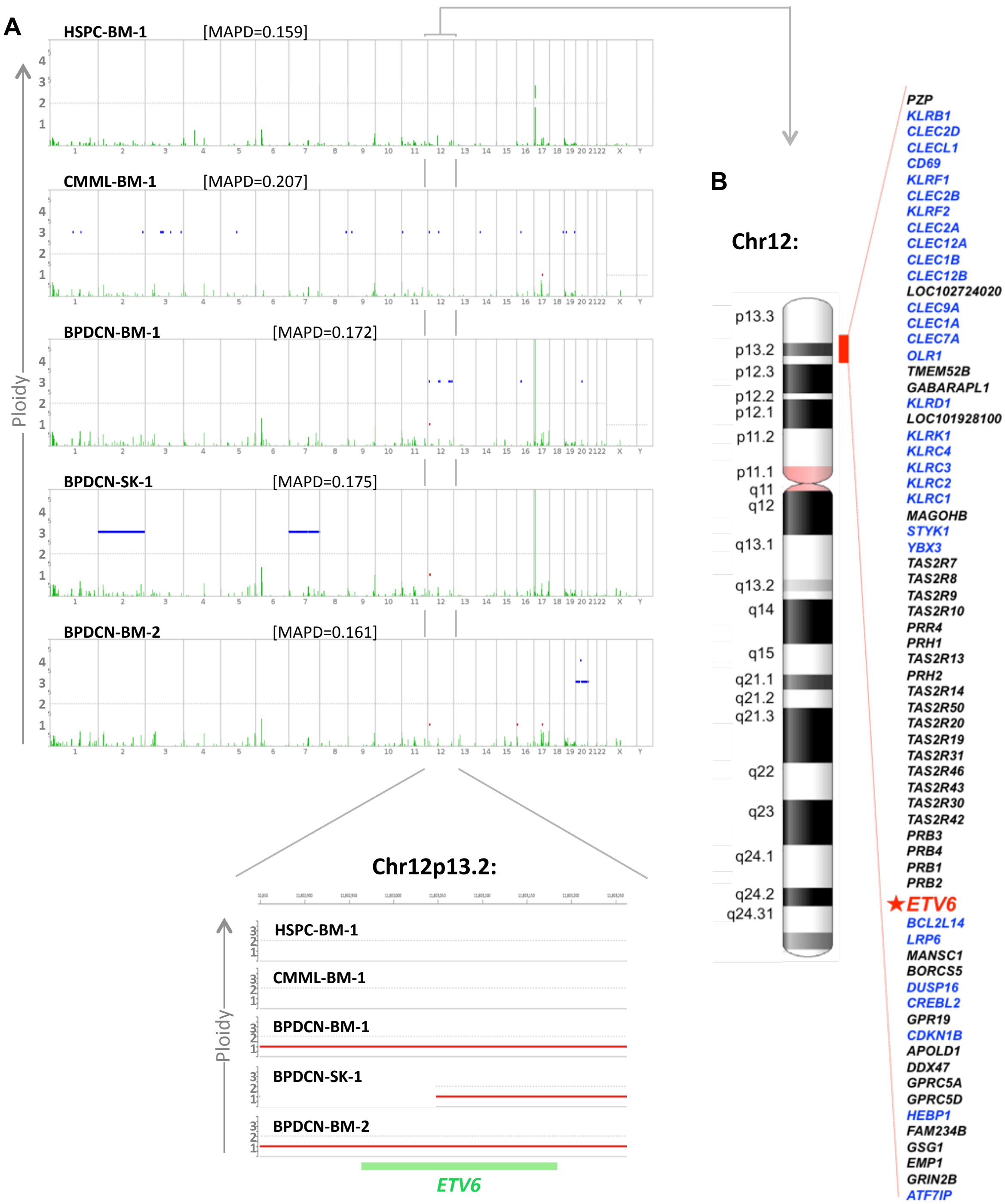

Supplemental Figure 4. A. Whole genome view indicating location and distribution of copy number variants across the five sequenced somatic exomes. Y-axis indicates derived ploidy level; blue lines indicate regions of copy gain; red lines indicate regions of copy loss. The deletion spanning *ETV6* is magnified in the inset, confirming *ETV6* loss exclusively in the three BPDCN samples. **B.** Segment on chromosome 12p listing all genes within the region deleted across the BPDCN samples. Along with *ETV6* (marked in red), all genes with documented roles with links to biology/function of dendritic cells and/or oncogenesis are highlighted in blue.

A

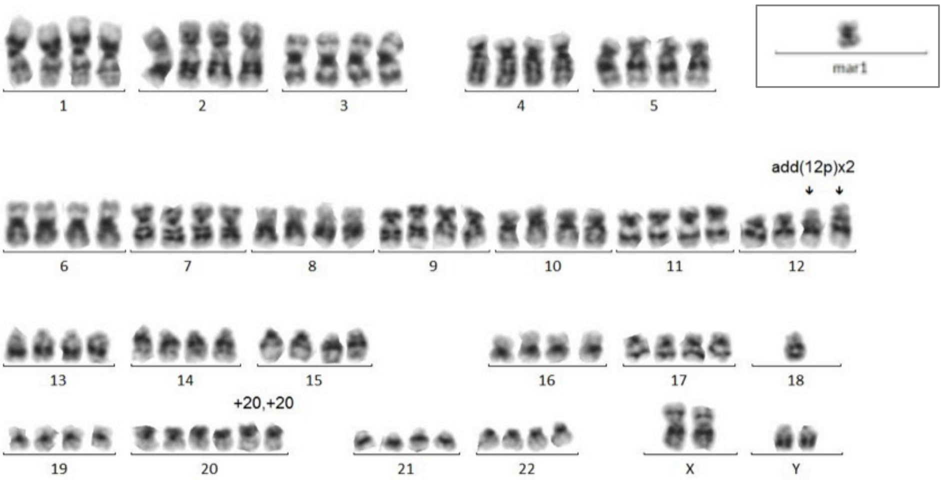

B

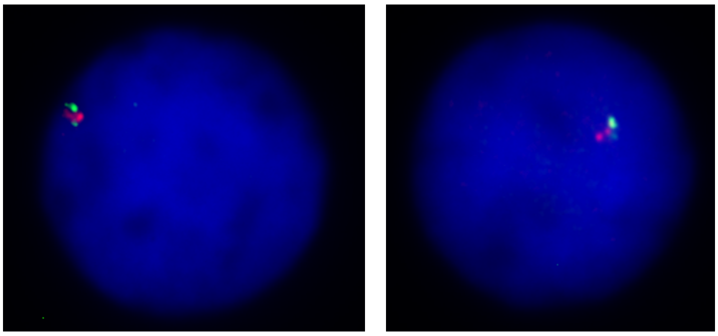

Supplemental Figure 5. A. Karyogram from bone marrow at disease progression post three cycles azacitidine, confirming the indicated abnormalities (including abn12p including deletion of *ETV6*) on an abnormal tetraploid background. An unidentified marker chromosome is displayed in the inset. **B**. Fluorescence in-situ hybridization using an *ETV6* break-apart probe, showing a single unrearranged copy of *ETV6* in exemplar cells. While all metaphase preparations were near tetraploid (in which monoallelic *ETV6* loss would be expected to result in 2 *ETV6* copies indistinguishable from the normal diploid state by interphase FISH), 59/100 interphase cells displayed a single unrearranged *ETV6* pattern. It was not possible to establish conclusively whether these represented near-diploid cells with single copy *ETV6* loss, or near-tetraploid cells with an addition 3rd *ETV6* deletion; a near-tetraploid metaphase was observed with only a single *ETV6* signal, indicating the latter as most likely.

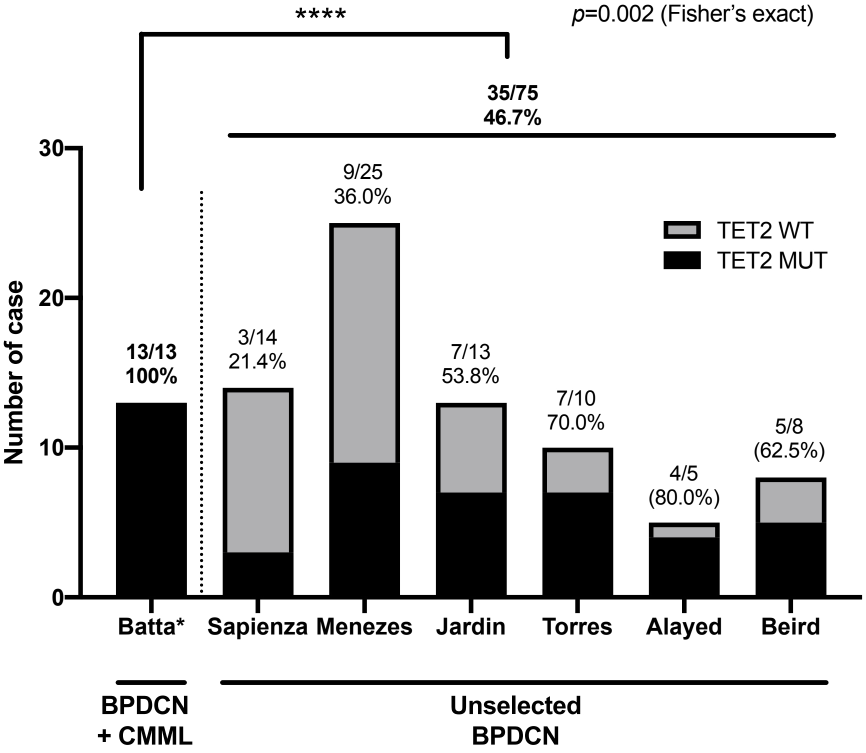

Supplemental Figure 6. Frequency of *TET2* mutations comparing the cohort of BPDCN + CMML cases identified in this study (Supplemental Table 3) against six published cohorts of unselected BPDCN patients sequenced for *TET2* mutations (labelled by first author name)[5-10]. *TET2* mutated patients are shaded black, stacked with *TET2* wild-type patients shaded grey; proportion of *TET2* mutant cases for each cohort is indicated above each bar. p value is derived by Fisher's exact test comparing the current cohort versus the combined unselected BPDCN cases.

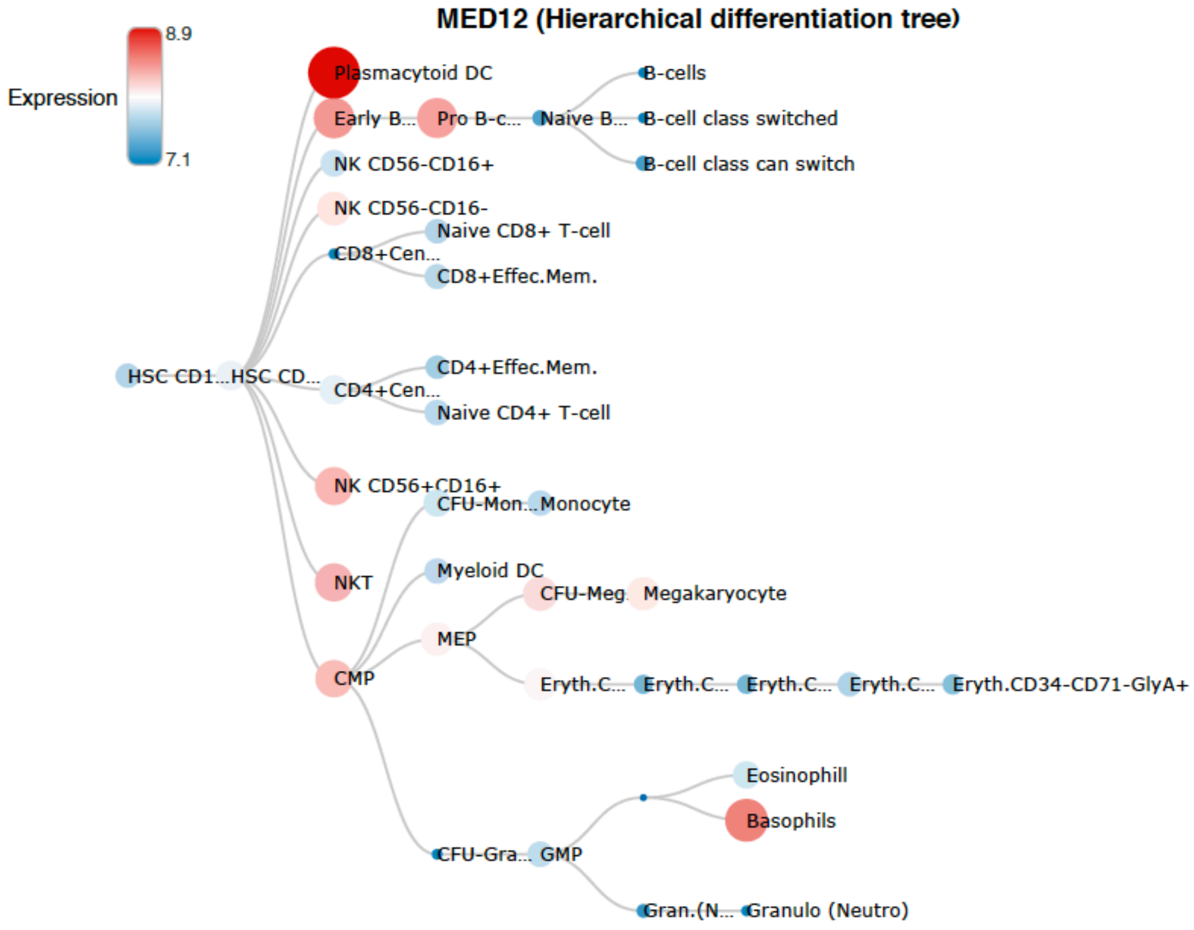

Supplemental Figure 7. Hierarchical differentiation tree showing *MED12* expression across normal hematopoiesis as highest in plasmacytoid dendritic cells. Derived from the DMAP dataset (n=211; source accession GSE24759)[11]; plot taken from BloodSpot[12] (http://servers.binf.ku.dk/bloodspot/); accessed 14 November 2020.

Index Case: MED12 G44S

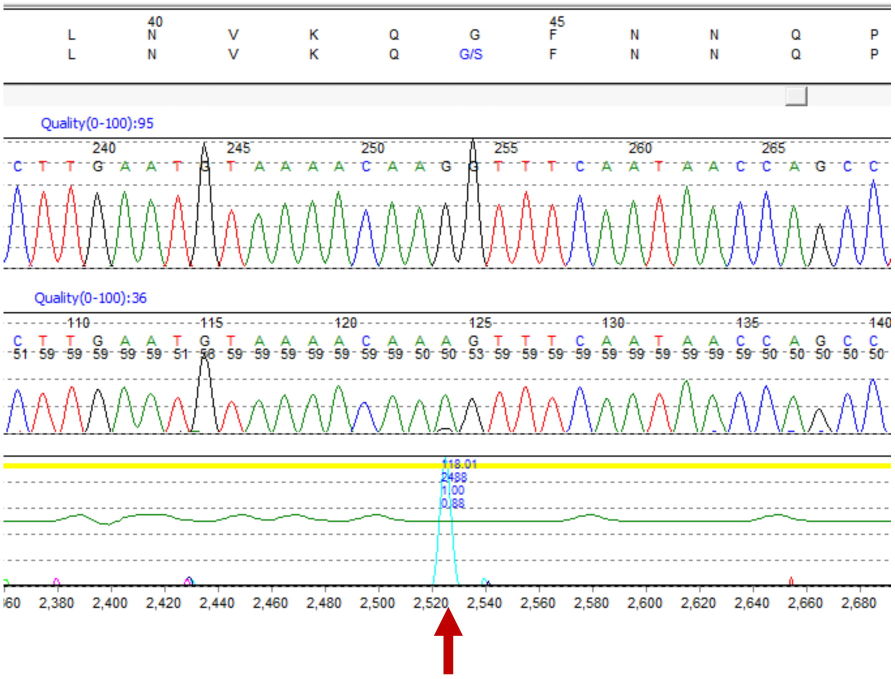

Example MED12-WT Case

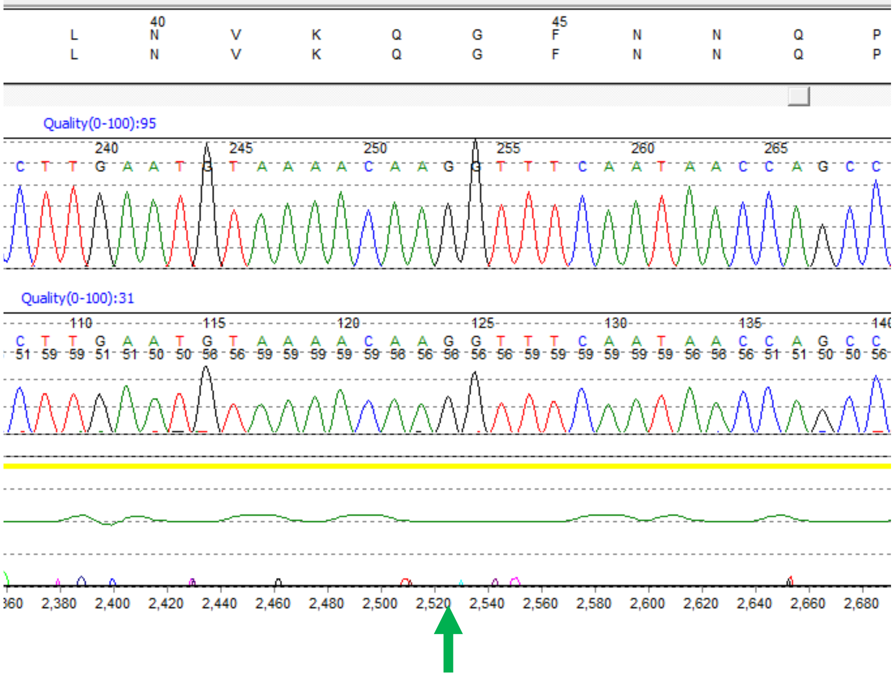

Supplemental Figure 8. Sanger sequencing validation of the *MED12* p.Gly44Ser mutation site in genomic DNA from the presentation skin biopsy specimen in the index case (upper) and an exemplar MED12 wild-type patient (lower).

Supplemental Table 1. Selected Quality Control metrics for the six samples subjected to whole exome sequencing (IonTorrent AmpliSeq Exome) in this study.

SAMPLE NAME	HSPC-BM-1	CMML-BM-1	BPDCN-BM-1	BPDCN-SK-1	BPDCN-BM-2	GERMLINE
**Source**	**BM**	**BM**	**BM**	**Skin**	**BM**	**Buccal**
**Timepoint**	**Diagnosis**	**Diagnosis**	**Diagnosis**	**Diagnosis**	**Progression**	**Diagnosis**
**Total Reads**	81,805,826	82,954,998	65,099,852	79,993,141	83,291,209	41,464,047
**Mean Coverage Depth** (fold)	**267.7x**	**275.6x**	**215.3x**	**254.4x**	**271.8x**	**132.8x**
**Coverage within Target Region**	99.7%	99.7%	99.7%	99.7%	99.7%	99.7%
**MAPD score** (Median Absolute Pair-wise Difference)	0.345	0.372	0.374	0.317	0.346	0.394

Supplemental Table 2. All somatic variants identified by whole exome sequencing across the five (non-germline) samples from the patient in this study. Samples in which the mutation was present, whether the variant has been documented in COSMIC, pathogenicity prediction according to Varsome, and Cluster number (from those variants passing coverage threshold for SciCLone/ClonEvol analysis) are provided. Genomic coordinates related to reference genome hg19; SNV: single nucleotide variant; Indel: insertion/deletion; MNV: multiple nucleotide variant.

See MS Excel file

Supplemental Table 3. Cohort of published and unpublished cases of concomitant BPDCN and CMML with selected clinical, cytogenetic and molecular metadata. #: unpublished case; ?: unknown; nd: not done; ns: not stated; BM: bone marrow; LN: lymph node; Bx: biopsyT: targeted sequencing; WES: whole exome sequencing; D: diagnosis.

**Reference**	**Location**	**Sex**	**Age (at BPDCN)**	**BPDCN (:CMML) Chronology**	**BPDCN site/s**	**Karyotype *(at BPDCN Diagnosis)***	**Mutation Profile *(at BPDCN Diagnosis)***	**VAF**		**Sequencing**	
								**BM MNC**	**Skin Bx**	**Method**	**Timepoints**
***Current study**	Christie (UK)	M	73	Secondary (+ 10 years)	Skin; BM	46,XY	**TET2 E1010X**<br>ZRSR2 771+2T>C<br>*+ others (Table S1)*	44%<br>45%<br>*Table S1*	50%<br>90%<br>*Table S1*	T (27 genes); WES	D [BPDCN] → Progression [BPDCN]
**Hu[13]**	MDACC (USA)	M	64	Concurrent	Skin; BM	Not stated	**TET2 (unspecified)**	ns	-	T (ns)	D [CMML+ BPDCN]
**Brunetti[14]**	Perugia (Italy)	M	82	Secondary (+ 2 years)	Skin	46,XY	**TET2 Y1244fs*1266**<br>**TET2 Q810X**<br>SRSF2 P95H<br>JAK2 V617F	50%<br>49%<br>46%<br>15%	38%<br>34%<br>37%<br>wt	T (54 genes)	D [BPDCN]
**Patnaik[15]**	Mayo (USA)	M	64	Secondary (+ 3 years)	Skin; BM	46,XY	**TET2 G523fs***<br>SRSF2 P95L<br>PHF6 Q251H<br>PLP1 T116M<br>PLCXD3 T282M<br>TRMT61B Y219C<br>STK3 R178fs*<br>SLC25A10 R263C<br>DIP2A R309W<br>SARDH G641E<br>IVL 227_236del<br>RB1 R251X<br>CROCC T1532S<br>ERCC4 V870I<br>CHP2 R34Q<br>RB1 *CNV*	57 → 77%<br>37 → 38%<br>57 → 78%<br>74 → 91%<br>39 → 47%<br>20 → 47%<br>42 → 42%<br>32 → 41%<br>35 → 40%<br>19 → 34%<br>65% → wt<br>wt → 68%<br>wt → 44%<br>wt → 39%<br>wt → 32%<br>NO → YES	-<br>-<br>-<br>-<br>-<br>-<br>-<br>-<br>-<br>-<br>-<br>-<br>-<br>-<br>-<br>-	WES	D [CMML] → BPDCN transformation

**Suma[16]**	Tsukuba (Japan)	M	65	Concurrent	Skin	46,XY	**TET2 K1005fs***<br>**TET2 D1129fs***<br>SRSF2 P95H<br>NPM1 L287fs*	46 → 47%<br>wt → 31%<br>47%<br>wt	42%<br>40%<br>32%<br>44%	T (67 genes)	D [BPDCN] → Relapse [BPDCN]
**Lebecque[17]**	Bordeaux (France)	M	74	Secondary (+ 2 years)	Skin; LN; BM	46,XY	nd	nd	nd	nd	nd
**Sukegawa[18]**	Tsukuba (Japan)	?	?	Primary	Skin; LN; BM	?	**TET2 (unspecified)**	ns	ns	T (ns)	D [BPDCN]
**Li[19]**	Mayo (USA)	M	68	Secondary (+ 4 years)	Skin	46,XY	**TET2 Q180Rfs*3**<br>**TET2 G1152X**<br>ZRSR2 c.312+1G>T<br>CDKN2A *CNV*<br>CDKN2B *CNV*<br>MTAP *CNV*	26%<br>27%<br>81%<br>no<br>no<br>no	38%<br>33%<br>75%<br>YES<br>YES<br>YES	T (ns)	D [BPDCN]
**Espasa[20]**	Barcelona (Spain)	M	87	Concurrent	Skin; BM	46,XY	**TET2 M1333N**<br>ZRSR2 N249T<br>ASXL1 Q882X	ns<br>ns<br>wt	ns<br>ns<br>ns	T (32 genes)	D [CMML+ BPDCN]
**Luskin[21]**	Dana Farber (USA)	M	78	Secondary (+ 10 years)	Skin; BM	46,XY	**TET2 C1642Sfs*53**<br>**TET2 C1811Vfs*9**<br>SRSF2 P95H<br>ASXL1 E635Rfs*15<br>CREBBP N1734Ifs*8<br>CSF3R T618I<br>NRAS G12D<br>NRAS G12V<br>NRAS G12S<br>RUNX1 F116S<br>Multiple CNVs (del)	51 → 50%<br>53 → 53%<br>55 → 56%<br>29 → 27%<br>50 → 36%<br>9 → 15%<br>7 → 9%<br>14 → 9%<br>3 → 1%<br>46 → 33%<br>ns → ns	- → 46%<br>- → 44%<br>- → 44%<br>- → ns<br>- → ns<br>- → ns<br>- → ns<br>- → ns<br>- → ns<br>- → ns<br>- → YES	T (95-genes → 282 genes)	D [BPDCN+CMML>AML] → Progression [BPDCN]
**Vitte[22]**	FSGCL cohort (France)	F	88	Concurrent	Skin; ?other	ns	nd	nd	nd	nd	nd
**Vitte[22]**	FSGCL cohort (France)	M	69	Concurrent	Skin; ?other	46,XY	nd	nd	nd	nd	nd

**Vitte**[22]	FSGCL cohort (France)	M	60	Secondary (+ ns)	Skin; ?other	ns	nd	nd	nd	nd	nd
**Vitte**[22]	FSGCL cohort (France)	M	74	Secondary (+ ns)	Skin; ?other	ns	nd	nd	nd	nd	nd
**#**	Christie (UK)	F	77	Concurrent	Skin	46,XX	**TET2 Q361X**<br>**TET2 S689Pfs*11**<br>**TET2 I1139Mfs*9**<br>DNMT3A R882H<br>NPM1 W288Cfs*12	22%<br>12%<br>43%<br>41%<br>13%	-<br>-<br>-<br>-<br>-	T (40 genes)	D [BPDCN + CMML>AML]
**#**	Christie (UK)	M	72	Concurrent	Skin, LN, BM	46,XY	**TET2 P612Lfs*27**<br>**TET2 N767Kfs*2**<br>NRAS G12D	38%<br>36%<br>11%	-<br>-<br>-	T (40 genes)	D [BPDCN + CMML]
**#**	Mayo (USA)	M	73	Concurrent	Skin; BM	46,XY	**TET2 W1255X**<br>**TET2 M804Rfs*9**<br>ASXL1 G646Wfs*12<br>ZRSR2 ns<br>ZRSR2 ns	69%<br>13%<br>36%<br>81%<br>92%	-<br>-<br>-<br>-<br>-	T (ns)	D [BPDCN + CMML]
**#**	MSKCC (USA)	M	66	Secondary (+ ns)	Skin	46,XY	nd	nd	nd	nd	nd
**#**	MSKCC (USA)	M	77	Secondary (+ ns)	Skin; LN; BM	43-44,XY,del(5)(q13q33),inv(6)(p11q23),-9,add(11)(p15),-12,der(21)(t(12;21)(q13;p11.1),-22	nd	nd	nd	nd	nd
**#**	MSKCC (USA)	F	66	Secondary (+ 5 years)	Skin; BM	46,XX	**TET2 S817Ifs*5**<br>DNMT3A A791Pfs*11<br>ETV6 X110_splice<br>*Multiple CNVs (del) inc ETV6*	96%<br>43%<br>45%<br>NO	84%<br>39%<br>0%<br>YES	T (400 genes)	D [CMML] → BPDCN transformation

Supplemental References

1. Greystoke BF, Huang X, Wilks DP, Atkinson S, Somervaille TCP. Very high frequencies of leukaemia-initiating cells in precursor T-acute lymphoblastic leukaemia may be obscured by cryopreservation. *Brit J Haematol* 2013; **163**(4)**:** 538-541.

2. Kopanos C, Tsiolkas V, Kouris A, Chapple CE, Aguilera MA, Meyer R*, et al.* VarSome: the human genomic variant search engine. *Bioinformatics* 2019; **35**(11)**:** 1978-1980.

3. Miller CA, White BS, Dees ND, Griffith M, Welch JS, Griffith OL*, et al.* SciClone: Inferring Clonal Architecture and Tracking the Spatial and Temporal Patterns of Tumor Evolution. *PLoS Comput Biol* 2014; **10**(8)**:** e1003665.

4. Dang HX, White BS, Foltz SM, Miller CA, Luo J, Fields RC*, et al.* ClonEvol: clonal ordering and visualization in cancer sequencing. *Ann Oncol* 2017; **28**(12)**:** 3076-3082.

5. Sapienza MR, Abate F, Melle F, Orecchioni S, Fuligni F, Etebari M*, et al.* Blastic plasmacytoid dendritic cell neoplasm: genomics mark epigenetic dysregulation as a primary therapeutic target. *Haematologica* 2019; **104**(4)**:** haematol.2018.202093.

6. Menezes J, Acquadro F, Wiseman M, Gómez-López G, Salgado RN, Talavera-Casañas JG*, et al.* Exome sequencing reveals novel and recurrent mutations with clinical impact in blastic plasmacytoid dendritic cell neoplasm. *Leukemia* 2014; **28**(4)**:** 823-829.

7. Jardin F, Ruminy P, Parmentier F, Troussard X, Vaida I, Stamatoullas A*, et al.* TET2 and TP53 mutations are frequently observed in blastic plasmacytoid dendritic cell neoplasm. *Brit J Haematol* 2011; **153**(3)**:** 413-416.

8. Torres ANB, Cats D, Mei H, Fanoni D, Gliozzo J, Corti L*, et al.* Whole-genome analysis uncovers recurrent IKZF1 inactivation and aberrant cell adhesion in blastic plasmacytoid dendritic cell neoplasm. *Genes Chromosomes Cancer* 2020; **59**(5)**:** 295-308.

9. Alayed K, Patel KP, Konoplev S, Singh RR, Routbort MJ, Reddy N*, et al.* TET2 mutations, myelodysplastic features, and a distinct immunoprofile characterize blastic plasmacytoid dendritic cell neoplasm in the bone marrow. *Am J Hematol* 2013; **88**(12)**:** 1055-1061.

10. Beird HC, Khan M, Wang F, Alfayez M, Cai T, Zhao L*, et al.* Features of non-activation dendritic state and immune deficiency in blastic plasmacytoid dendritic cell neoplasm (BPDCN). *Blood Cancer J* 2019; **9**(12)**:** 99.

11. Novershtern N, Subramanian A, Lawton LN, Mak RH, Haining WN, McConkey ME*, et al.* Densely Interconnected Transcriptional Circuits Control Cell States in Human Hematopoiesis. *Cell* 2011; **144**(2)**:** 296-309.

12. Bagger FO, Sasivarevic D, Sohi SH, Laursen LG, Pundhir S, Sønderby CK*, et al.* BloodSpot: a database of gene expression profiles and transcriptional programs for healthy and malignant haematopoiesis. *Nucleic Acids Res* 2016; **44**(D1)**:** D917-D924.

13. Hu Z, Sun T. Blastic plasmacytoid dendritic cell neoplasm associated with chronic myelomonocytic leukemia. *Blood* 2016; **128**(12)**:** 1664-1664.

14. Brunetti L, Battista VD, Venanzi A, Schiavoni G, Martelli MP, Ascani S*, et al.* Blastic plasmacytoid dendritic cell neoplasm and chronic myelomonocytic leukemia: a shared clonal origin. *Leukemia* 2017; **31**(5)**:** 1238-1240.

15. Patnaik MM, Lasho T, Howard M, Finke C, Ketterling RL, Al-Kali A*, et al.* Biallelic inactivation of the retinoblastoma gene results in transformation of chronic myelomonocytic leukemia to a blastic plasmacytoid dendritic cell neoplasm: shared clonal origins of two aggressive neoplasms. *Blood Cancer J* 2018; **8**(9)**:** 82.

16. Suma S, Sakata-Yanagimoto M, Nguyen TB, Hattori K, Sato T, Noguchi M*, et al.* Blastic plasmacytoid dendritic cell neoplasm arising from clonal hematopoiesis. *Int J Hematol* 2018; **108**(4)**:** 447-451.

17. Lebecque B, Vial JP, Pigneux A, Lechevalier N. Chronic myelomonocytic leukaemia followed by blastic plasmacytoid dendritic cell neoplasm. *Brit J Haematol* 2019; **185**(3)**:** 398-398.

18. Sukegawa S, Sakata-Yanagimoto M, Matsuoka R, Momose H, Kiyoki Y, Noguchi M*, et al.* [Blastic plasmacytoid dendritic cell neoplasm accompanied by chronic myelomonocytic leukemia successfully treated with azacitidine]. *Rinsho Ketsueki* 2019; **59**(12)**:** 2567-2573.

19. Li M, Shah M, Binder M, Lasho T, Carr R, Mangaonkar A*, et al.* Cutaneous blastic plasmacytoid dendritic cell neoplasm arising in the context of TET2 and ZRSR2 mutated clonal cytopenias of unknown significance, secondary to somatic copy number losses involving CDK2NA/2NB and MTAP. *Am J Hematol* 2019; **95**(2)**:** E31-E34.

20. Espasa A, Sorigue M, Tapia G, Cabezon M, Vergara S, Raya M*, et al.* Chronic myelomonocytic leukemia and blastic plasmacytoid dendritic cell neoplasm. A case report and systematic review. *Cytom Part B Clin Cytom* 2020.

21. Luskin MR, Kim AS, Patel SS, Wright K, LeBoeuf NR, Lane AA. Evidence for separate transformation to acute myeloid leukemia and blastic plamacytoid dendritic cell neoplasm from a shared ancestral hematopoietic clone. *Leukemia Lymphoma* 2020; **61**(9)**:** 1-4.

22. Vitte F, Fabiani B, Bénet C, Dalac S, Balme B, Delattre C*, et al.* Specific Skin Lesions in Chronic Myelomonocytic Leukemia: A Spectrum of Myelomonocytic and Dendritic Cell Proliferations: A Study of 42 Cases. *Am J Surg Pathology* 2012; **36**(9)**:** 1302-1316.
